# Effects of high-performance human resource practices in the education sector: The mediational model

**DOI:** 10.3389/fpsyg.2022.1042082

**Published:** 2022-12-21

**Authors:** Cunbo Yang, Fakhra Yasmin

**Affiliations:** ^1^School of Management, Zhengzhou Shengda University, Zhengzhou, China; ^2^Graduate School, Claro M. Recto Academy of Advanced Studies, Lyceum of the Philippines University, Manila, Philippines; ^3^School of Education, South China Normal University, Guangzhou, China; ^4^Department of Informatics and Quantitative Methods, Faculty of Informatics and Management, University of Hradec Kralove, Hradec Králové, Czech Republic

**Keywords:** high-performance human resource practices, job stress, turnover intentions, person–organization fit, person–job fit

## Abstract

In order to better understand the link between High-Performance Human Resource Practices (HPHRPs) and outcomes, this study examines the mediating roles of person–job fit (PJ fit) and person–organization fit (PO fit) using congruence theory. Through a survey questionnaire, data were gathered from 296 people who work at educational institutions in China. The results demonstrated that the association between HPHRPs and outcomes is mediated by both PJ fit and PO fit. We observed theoretical implications and discovered that HPHRPs are an important antecedent that builds congruence among employees' values and goals and organizational values and goals, as well as their job goals, which in turn prevents employees from experiencing stress and developing intentions to leave their workplace. The current study adds to extant literature on education and HPHRPs by identifying PJ fit and PO fit as mechanisms through which HPHRPs demonstrate their authority on employee outcomes. The managerial implications, limitations, and directions for future studies are included in detail at the end.

## Introduction

In contemporary work environments, the ability to attract, recruit, and retain talented employees are a prerequisite for a company's success. These factors also form employee perspectives, influencing their selection of an appropriate profession and employer, which are crucial for achieving a higher quality of work life (Alniacik et al., 2013). High-performance human resource practices (HPHRPs) are, for the most part, seen as an arrangement of interconnected HPHRPs intended to upgrade employees' value and execution within corporations (Messersmith et al., 2011). Most researchers have analyzed the relationship between HPHRPs and employees' work outcomes (Alqudah et al., 2022). Despite having adequate knowledge of HPHRPs, researchers still believe that there are unexplored links between HPHRPs and job outcomes that need to be explored (Hauff et al., 2022). We also need to further explore more intervening variables to better explain the HPHRPs and outcome links (Murphy et al., 2018).

Organizational behavior researchers have recently started to examine several underlying mechanisms to better understand and explain the relationships between HPHRPs and outcomes. For instance, human resource wellbeing attribution and human resource performance attribution explain the link between HPHRPs, commitment, and job strain (Van De Voorde and Beijer, 2015). Similarly, job embeddedness, the organizational climate, employee engagement, availability at work, meaningfulness, psychological experiences of safety, job demands, job resources, and public service motivation are among a few other mediating mechanisms that have been thought to explain the link between HPHRPs and job outcomes (Hauff et al., 2022). However, to date, no consideration has been given to the role of job and organizational congruence processes and the relationships between HPHRPs and outcomes.

By exploring congruence theory (Holland, 1997), we have identified gaps related to two important yet understudied mediators, person–organization fit (PO fit) and personal job fit (PJ fit). These particular mediators might provide further insights into HPHRPs and job outcomes above and beyond other mediators. Holland (1997) believed that employees desire an environment that is highly congruent with their personalities and individual values, and that this congruence leads to positive results. We believe that HPHRPs develop PO fit and PJ fit, which decrease job outcomes, specifically turnover intention (TOIs) and job stress. HPHRPs are believed to create synergy, and because of this, employees feel that the workplace is congruent with their own objectives, job requirements, and organization. This congruence ultimately leads to positive outcomes in the form of a decrease in TOIs and job stress.

The reason for considering both these variables is that we believe that HPHRPs develop congruence between an individual's beliefs and their job, as well as the organization, and both are important for reducing TOIs and job stress. Various deliberations provoked the selection of variables in the current research. The PO fit and PJ fit are viewed in relation to their outcomes by current employees in an organization (Haider et al., 2022). HRPs in the organization can improve the intensity of employees' PO and PJ fits and, as a result, can change employees' behaviors and attitudes. Previous studies reveal that work-related stress and TOIs are notably connected with HPHRPs, PO, and PJ fit (Chen et al., 2022; Pattanawit and Charoensukmongkol, 2022). In addition, job stress and TOIs are crucial for companies that have serious concerns about their performance and employees (Syed et al., 2021). Findings from previous studies show that HPHRPs can positively affect employees by improving the similarities between their job and their existing company (Mostafa, 2017).

It is pertinent to mention that PJ fit is different from PO fit (Gould-Williams and Mohamed, 2010). We have tried to propose PO fit and PJ fit due to the difference in their scopes. The former is broader in scope and entails the congruence between personal and organizational values. In contrast, the latter explains a narrow scope of congruence that encompasses similarities with job-related values. Previous research indicates that PO fit and PJ fit hinder intentions toward turnover and related work issues (Junaedi and Wulani, 2021). Therefore, this study encompasses both narrow and broader scopes of congruence as an underlying mechanism between HPHRPs and outcomes.

This research intends to address this query by evaluating the effect of PO fit and PJ fit as mediators between HPHRPs and outcomes in the current organization. This will fulfill the requirement raised by researchers to carry out a study on these two variables, PO fit (Paauwe et al., 2013) and PJ fit (Mashhadi et al., 2015), as mediators between HPHRPs and employees' approaches.

The present study will add value to the literature by describing the mediating mechanisms of PO and PJ fits in the relationship between HPHRPs and job outcomes through the lens of person–environment congruence theory (1997). It also helps the manager understand the role of HPHRPs in an organizational setting and their impact on job outcomes such as employee TOIs and stress. It provides insights into the role of PO fit and PJ fit in an organizational setting.

We contribute to the existing body of knowledge by identifying two important mediators between HPHRP-outcome relations and explaining this relationship with the help of congruence theory. This theory emphasizes the need to increase congruence among employees and their organizations and jobs to yield positive outcomes. In other words, HPHRPs give employees the feeling that their goals are congruent with organizational goals and their job. Employees who feel this alignment in values and goals are less likely to experience stress and tend to stay with the organization.

## Theory and hypotheses development

The current study was conducted in accordance with person–environment congruence theory (Holland, 1997). The concept of congruence was pioneered by Parsons (1909); however, he discussed this phenomenon's social and cultural implications. Holland (1959) presented a more comprehensive view of person–environment fit, giving rise to congruence theory, which is still widely applied in psychology, business, and other domains. Holland (1997) used the word congruence to explain the level of alignment between the individual and their environment. They further explained that higher person–environment congruence or agreement leads to positive results and vice versa. In other words, congruence refers to the degree of synchronization between the individual and their occupational environments, such as their organization and job. This study, therefore, proposed that individuals always strive for a congruent environment. Individuals who achieve a congruent environment are more likely to show positive outcomes and avoid negative behaviors such as TOIs and stress.

Holland (1997) also suggested that employers attempt to create an environment that causes congruency by eliminating the individuals who do not fit in and supporting those who do. Keeping this theory in mind, we propose that the HPHRP environment, due to the use of a bundle of HR practices, increases congruence between the environment and individuals in the form of PJ fit and PO fit, which leads to a decrease in job stress and TOIs; this is because employees are more likely to stay in an environment that offers greater congruence with their personalities.

### HPHRPs

The privatization/deregulation scenarios, the competitive climate, and technological advancements have forced management to recalibrate numerous HR and other management practices in the context of the rapidly changing global economy. Organizations are now required to use HPHRPs that increase competitive advantages due to environmental changes (Gurbuz, 2009). Many scholars have identified HPHRPs in the literature. HPHRPs enhance employee competency, level of expertise, and aptitude, as well as create an opportunity to improve the company's output through a knowledge-sharing environment (López et al., 2004; Wei et al., 2012). HPHRPs are also defined as a bundle of HR practices adopted by any organization to achieve positive outcomes (Beltrán-Martín and Bou-Llusar, 2018). Recent research by Posthuma et al. (2013) identified 61 HRPs being practiced as HPHRPs. Despite wide literature on HPHRPs, the literature has a dearth in respect of types of human resource practices.

The current research used five HR practices in order to measure the employee's sensitivity to HPHRPs. The practices used are the most popular while analyzing the relationship between HPHRPs and employees' job-related outcomes (Alqudah et al., 2022; Hauff et al., 2022). Furthermore, these practices are believed to be high predictors of PJ and PO fit. Thus, we considered job security, promotion, autonomy at work, training/development, and communication HRPs. Here, it is important to mention that we took them collectively and not separately because HPHRPs create synergy by combining a bundle of HR practices.

Research in the past has found that PO/PJ fit awareness has a major effect on outcomes related to work (Pattanawit and Charoensukmongkol, 2022). Additionally, according to Cable and DeRue (2002), different fit types may be examined, as they can affect various work-related outcomes differently. Few researchers have also studied PO/PJ fits jointly. Available research has examined whether employees can differentiate between various fits (Chen et al., 2022). The focal point of our study will be the effect of PO/PJ fits on work stress and the TOIs of current employees in organizations.

### HPHRPs and PO fit

Our research evaluated the similarity between employees and company missions and objectives, focusing on the similarity between employee personalities and those of their employers. Schneider's (1987) ASA system clarifies how HPHRPs may influence the fit between employees and their employers. The primary reason behind this structure is that people are drawn to various sorts of companies because of their pre-passage perception of the company's core qualities and objectives. At that point, companies pick people who fit their qualities and objectives through formal and informal selection methods. In the long term, a few representatives may choose to leave because their qualities and objectives may change or no longer match those of the company. The PO fit is “progressive as well as adaptable” because people adjust to companies, and companies change after some time (Petrides and Furnham, 2001). Therefore, contracting practices are essential for assessing a person's ability to cope with the company, and different HPHRPs are instrumental in helping employees coordinate with their companies. For example, in preparing and advancing employees, work-related security, stability, and advancement impart authoritative qualities, objectives, and desires to employees, which ought to expand employees' impressions of PO fit (Akhtar et al., 2020). Two reviews assessed the association between HPHR practices and PO fit, demonstrating that employees' perceptions of HPHRPs coincided with those of companies (Uppal, 2020). Although most reviews have analyzed the impacts of PO fit on worker outcomes as a part of the HPHRP (Narayanan and Sekar, 2009). Person–environment congruence theory also claims that when the organization takes care of the employees by adopting HPHRPs for their benefit, employees start to feel that there is a high degree of congruence between them and their organization. Thus,

*H1: HPHRPs is positively related to PO fit*.

### Mediating role of PO fit

The most extensively examined type of fit is PO fit since it has been recognized that it can significantly influence behavioral outcomes (Akhtar et al., 2020). Numerous studies have focused on how PO fit is an underlying mechanism in the link between service motivation and outcomes (Hue et al., 2022). Generally speaking, stress occurs when a person realizes that the demands of a situation exceed their capacity to deal with them (Mansoor et al., 2011). Stress inside the work environment is associated with employment stress, a heavy workload, or work-related anxiety (Kalia, 2002). Little consideration has been given to the connection between HPHRPs and work results that weaken worker wellbeing and prosperity, for example, job stress (Jensen et al., 2011). Gould-Williams and Mohammed (2021) found that HPHRPs adversely influenced work stress.

It has been suggested that occupation stress results from an absence of compatibility between representative and authoritative qualities (Edwards et al., 1990). At the end of the day, work stress, for the most part, increases when the company's qualities differ from those of the employee. This distinction creates an absence of fit, which, as a result, causes negative mental impacts (Edwards et al., 1990). However, a raised PO fit level shows the compatibility between employees and company qualities (Akhtar et al., 2020; Pattanawit and Charoensukmongkol, 2022). This compatibility makes it easy for employees to talk with their colleagues and seek their help, which will most likely cause diminished levels of occupational stress (Edwards et al., 1990).

Similarly, past research found the mediating effect of PO fit on employee behaviors (Uppal, 2020). Holland (1997) also believed that high person-organization congruence reduces negative behavior among employees. On this premise, the following hypotheses are proposed:

*H2: PO fit has a negative effect on job stress*.
*H3: PO fit mediates the HPHRPs and job stress link*.

As indicated by Lambert and Hogan (2009), TOI is more critical from a business perspective than actual employee turnover. In the event that businesses can legitimately comprehend the antecedents of TOIs, they can introduce changes that decrease these intentions. When employees leave, the company has to bear the cost of contracting and preparing different representatives (Lambert and Hogan, 2009). TOIs are less demanding to quantify and foresee than actual employee turnover (Syed et al., 2021) and are a superior indicator of administration practice.

It has been confirmed through research that HPHRPs have a negative relationship with intentions to quit (Uppal, 2020). In any case, specialists contend that the procedures through which this relationship happens remain unverifiable (Kehoe and Wright, 2013).

Employees have demonstrated a greater propensity to remain with organizations that share their interests (Schneider, 1987). Researchers have discovered that employees' intentions to leave are reduced by a better PO fit (Abdalla et al., 2018). It can be contended that HPHRPs might impact TOI indirectly through a PO fit. In this way, the company's and its employees' values will reflect their characteristics. This will strengthen the bonds between employees and both their company and their colleagues, which will decrease the chance of employees leaving (Abdalla et al., 2018).

*H4: PO fit has a negative effect on TOI*.
*H5: PO fit mediates HPHRPs and TOI links*.

### HPHRPs and PJ fit

Studies in the past corroborate the fact that there is a relationship between PJ fit and employee behavior on the job (Uppal, 2020; Junaedi and Wulani, 2021). Fit plays a vital role in the attainment of business accomplishments. However, the PJ fit is a basic, critical idea for individuals with employment features. Undoubtedly, without a solid match of individuals with work requirements, HR issues like poor staff output, the number of people leaving their organization, absence from the place of duty, and some others may amplify (Mathis and Jackson, 2003).

PJ fit points out the commonalities among employees' learning, aptitudes/abilities, and employment prerequisites (Carless, 2005). The PJ fit is accomplished once a representative has the skillset commensurate with the job requirements or once employment addresses employees' issues (Kristof-Brown, 2000). The HPHRPs can play an essential role in coordinating representatives with their employment (accomplishing PJ fit) and with the company (accomplishing PO fit). Nevertheless, it cannot be anticipated that those who do not feel their work satisfies their desire to give back to society or that their employer upholds public ideals will feel a fit and, as a result, do better than other individuals (Pandey et al., 2008). Psychological contract exhibits that HRPs are genuine segments by which employees can comprehend the terms of their business (Rousseau and Greller, 1994). The demand and supply of employees and their fitness level will probably be influenced by the attributes of the companies (Akhtar et al., 2020), which are conveyed through HR practices. In addition, HRPs, for example, who select and train individuals, can coordinate the individuals with occupational prerequisites. HRPs may expand the level of PO fit and PJ fit by constantly conveying qualities, attributes, requests, and desires of the company to employees by giving assets to change or increment representatives' KSAs. In this way, we recommend that offering employees a steady arrangement of “superior” HR practices will probably cause an increase in their level of fit with their company. This is also aligned with the person–environment theory. Thus, we suggested the following hypothesis:

*H6: HPHRPs are positively related to PJ fit*.

### Mediating role of PJ fit

Investigations of work selection decisions have additionally reported on the impact of worker improvement and reward frameworks on employment choices. Bretz and Judge (1994) observed compensation level and advancement openings as noteworthy indicators of an employment decision. Cable and Judge (1996) found that compensation strategies are unequivocally identified with work-hunt choices. After the preliminary period of employment decision and selection, socialization practice helps to create PO and PJ fits amongst beginners/companies (Den Hartog and Verburg, 2004; Uppal, 2020; Chen et al., 2022; Pattanawit and Charoensukmongkol, 2022). Companies utilize improvement and reward practices to encourage the desired behavior out of employees and strengthen the harmony between employees and their company (Boon et al., 2007). On the whole, the finding recommends that different HRPs, including hiring, training, evaluation, and remuneration procedures, can influence PJ fit. We related HPHRPs with PO/PJ fits and anticipated that the higher the arrangement of HRPs, the higher the level of PO and PJ fits.

Studies continue to demonstrate connections between PJ fit and essential work states of mind and practices (Abdalla et al., 2018). The PJ fit is one of the vital predictors of the company's success. The PJ fit is a basic yet vital idea that includes coordinating the learning, aptitudes, and capacities of individuals with the qualities of occupations. Without a strong alignment between the individual and work demands, the probability of lower worker results, higher TOIs, absence, and other HR-related issues can rises (Mathis and Jackson, 2003; Abdalla et al., 2018; Haider et al., 2021, 2022).

A worker's objectives and qualities can be aligned with the company for which they work; however, there might be a lack of cooperation between the fundamental missions and objectives of their employment and their capacities or occupation inclinations. Siegrist (1996) exhibited the model that employees might be defied with stress if their endeavors are not adequately remunerated or perceived by the company. The company can show appreciation for employees by giving an extrinsic reward, a compensation raise, or a promotion in their profession. At the end of the day, a profession that is excessively hard, and does not also match hard work with the endeavors of employees and the rewards given, is damaging for workforce morale (Siegrist et al., 1990; Siegrist, 1996). Thus, we propose the following hypotheses:

*H7: PJ fit has a negative effect on job stress*.
*H8: PJ fit mediates HPHRPs and job stress*.

The opening and rewards provided by HRPs indoctrinate the perception that employees will be rewarded for a job well done (Ramsay et al., 2000). As a response, the employees make good decisions on their own that are beneficial for the organization without being instructed to do so, are more loyal, and have a passion for their work and their organization. Thus, HRPs can influence employee behaviors through a PJ fit. Nowadays, numerous companies are increasing compensation and giving extra advantages to employees to retain them (Gumbus and Johnson, 2003). Organizations are aware that retaining employees is useful for maintaining and sustaining a competitive edge (Youndt et al., 1996; Walker, 2001). Thus, HPHRPs can enhance employee retention (Arthur, 1994). Mercer (2005) specified in his study that if employees are rewarded well, they tend to remain with the company. The top management's contribution, responsive behavior, and providing new openings can be beneficial for retaining employees (Birt et al., 2004).

Hollenbeck (1989) asserted that organizations might have to face higher TOIs from employees with lower levels of PJ fit. Employees who experience a mismatch between their skills and abilities and those expected from their occupation may intend to leave their current work to find a better alternative (Wilk and Sackett, 1996). Similarities between the worker and their work and/or company ethos lead to better performance, as the employee experiences more satisfied feelings at work, and is more likely to have company loyalty, meaning there is a reduction in the tendency to turn over (Iplik et al., 2011).

Past reviews have demonstrated that PJ fit discernments significantly affect work-related results (Pattanawit and Charoensukmongkol, 2022). An exploration by Cable and DeRue (2002) on company human resources recommends analyzing both sorts of fit, as they might be connected with various results. The PJ fit results in a reduction in TOIs (Uppal, 2020). Some research has examined both PJ fits in a similar review. These studies examined whether individual selectors differentiate between the two fits when selecting individuals (Bretz and Judge, 1994; Cable and Judge, 1996). Current research emphasizes the impact of two types of fits: work-related stress and employee intention to leave the existing organization. Thus, PJ fit acts as a mediator between HPHRPs and TOIs, such that employees who report high HPHRPs also report low TOIs because they are comfortable in their work environments.

*H9: PJ fit has a negative effect on TOI*.
*H10: PJ fit mediates HPHRPs and TOI*.

## Research methodology

**Figure 1:**
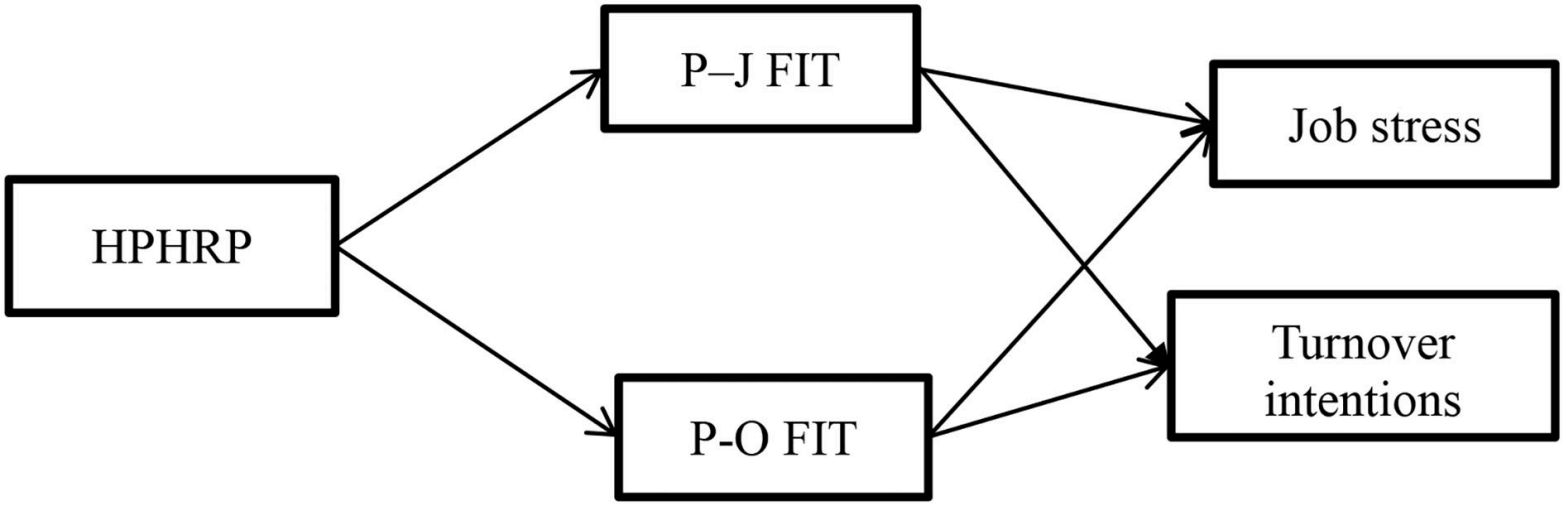
Research model.

Following the positivistic philosophy, the quantitative approach was used to test the previously discussed hypotheses using the model proposed in Figure 1. Employees served as the units of analysis, and data were collected through a questionnaire survey of education sector employees. The study was cross-sectional. In the same way that previous research has employed it, we used convenience sampling to obtain data from the education sector (Aslam et al., 2021; Ran et al., 2021, 2022; Syed et al., 2021; Yasmin et al., 2021; Zeb et al., 2021; Idrees et al., 2022). In order to check for common method bias (CMB), we checked a single Harmon factor, whose value was 32%. It indicated that CMB was not an issue.

Twenty items were used from earlier research (Den Hartog and Verburg, 2004) to measure *HPHRPs*. Sample items included “My institution provides excellent opportunities for personal skills development.” This measurement's Cronbach's alpha was 0.91. Four items were used to represent the PO fit developed by O'Reilly and Chatman (1986). A sample item was, “What this organization stands for is very important to me.” This measurement's Cronbach's alpha was 0.83. Four items were used to represent the PJ fit developed by Cable and Judge (1996). A sample item was, “To what degree do you think you possess the skills and abilities to perform this job?” (Cronbach's alpha is 0.80). *Job stress* was measured through four items introduced by Motowidlo et al. (1986). A sample item was “I almost never feel stressed at work” (Cronbach's alpha was 0.88). Four items were used to explore how the TOIs developed by Pfeffer and Jeffrey (1998). A sample item was “I would prefer another more ideal job to the one I have now” (Cronbach's alpha was 0.85).

For the identification of control variables, we conducted the one-way ANOVA test, and the result revealed that all demographic variables, i.e., gender, age, and education, had a significant effect on study variables. Therefore, we used any control variable while conducting further analysis.

## Results analysis

All employees received 365 surveys. There were 296 complete surveys received (response rate: 81%). Demographics included age, gender, and education. Most respondents were below the age of 36 (80%), 71 % were male, and 58 % had a master's degree.

The means and standard deviations of all variables were determined. The study variables were correlated. Table 1 shows that the HPHRPs were negatively correlated with the TOIs (−0.52^**^) and job stress (−0.62^**^) and positively correlated with the PO fit (0.68^**^) and PJ fit (0.66^**^). TOI was positively correlated with job stress (0.65^**^) and negatively correlated with the PO fit (−0.68^**^) and PJ fit (−0.63^**^). Job stress negatively correlated with the PO fit (−0.66^**^) and the PJ fit (−0.62^**^). Lastly, the PO fit positively correlated with the PJ fit (0.84^**^). The reliability of scales was examined through Cronbach's alpha. Reliability above 0.70 was considered significant (Guriting and Ndubisi, 2006).

**Table 1:** Correlation, descriptive statistic, and reliability.

	**Mean**	**Std. deviation**	**1**	**2**	**3**	**4**	**5**
HP	3.17	0.68	**(0.91)**				
TOI	2.67	0.91	−0.52^**^	**(0.85)**			
JS	2.70	1.10	−0.62^**^	0.65^**^	**(0.88)**		
PO	3.32	0.84	0.69^**^	−0.68^**^	−0.66^**^	**(0.83)**	
PJ	3.29	0.78	0.66^**^	−0.63^**^	−0.62^**^	0.65^**^	**(0.80)**

** Correlation is significant at 0.01 level (two-tailed).

N = 296. Alpha values are in brackets. Bold values are significant.

To test the mediation effect, we employed Preacher and Hayes (2004) process technique. According to Table 2 results, H1 and HPHRPs were positively related to the PO fit. The result showed that the HPHRPs positively affect the PO fit (*p* = 0.000 < 0.05, β = 0.91). Thus, H1 was substantiated. Similarly, according to H6, HPHRPs were positively related to the PJ fit. The result showed that the HPHRPs positively affect PJ fit (*p* = 0.000 < 0.05, β = 0.87). Thus, H6 was also substantiated.

**Table 2:** Mediation analysis.

**Effects**	**Coefficients**	**95% Confidence interval (CI)**
**Direct effect**		
HPHRP → P O Fit	0.91	[0.83, 1.15]
HPHRP → P J Fit	0.87	[0.72, 1.03]
P O Fit → Job Stress	−0.80	[−1.07, −0.53]
P O Fit → Turnover Intentions	−0.68	[−0.88, −0.64]
P J Fit → Job Stress	−0.51	[−0.84, −0.18]
P J Fit → TOIs	−0.65	[−0.94, −0.37]
**Indirect effect**		
HPHRP → Job Stress via P O Fit	−0.57	[−0.95, −0.18]
HPHRP → TOIs via P O Fit	−0.61	[−0.92, −0.45]
HPHRP → Job Stress via P J Fit	−0.45	[−0.77, −0.09]
HPHRP → TOIs via P J Fit	−0.57	[−0.84, −0.34]

n = 296. Bootstrap sample size = 5,000. LL, lower limit; CI, confidence interval; UL, upper limit.

The result showed that entering PO fit mediates HPHRPs and job stress. Because HRPs (*p* = 0.000, beta −0.57) negatively impacted job stress through PO fit, confidence interval values also validate the significance of the indirect effect (LL = −0.95, UL = −0.18). Thus, H2 and H3 were substantiated. The results revealed that under HPHRPs, employees' levels of PO fit increased, and their level of job stress decreased.

We found that the PO fit mediates between HPHRPs and TOIs. Because HR practices (*p* = 0.000, beta −0.61) negatively impacted TOI through PO fit, confidence interval values also validated the significance of the indirect effect (LL= −0.92, UL= −0.47). H4 and H5 were substantiated and the results revealed that under HPHRPs, employees' levels of PO fit increased, and their turnover intention reduced.

Results also showed that the PJ fit mediates HPHRPs and job stress. Because HPHRPs (*p* = 0.000, beta −45) negatively impact job stress through the PJ fit, confidence interval values also validate the significance of the indirect effect (LL = −0.77, UL = −0.09). So H7 and H8 were substantiated. Thus, the results reveal that under HPHRPs, employees' levels of PJ fit increased, and their job stress decreased.

According to the study results, the PJ fit mediates the HPHRPs and TOIs. Because HPHRPs (*p* = 0.000, beta −0.57) negatively impact TOI through PJ fit, confidence interval values also validate the significance of the indirect effect (LL = −84, UL = −0.34). Thus, H9 and H10 were substantiated. The results reveal that under HPHRPs, employees' levels of PJ fit increased, and their leaving intentions were reduced.

## Discussion

Recognizing the need for mediators between HPHRPs and employee outcomes to be examined, our study aimed to find a link between various types of fits and HRPs. The major reason behind choosing these two mediators was that there is little existing research on the role of PJ fit and PO fit between HPHRPs and outcome relationships. The existing literature also highlighted the need to identify the mediating mechanisms of the HPWS-outcome relationship, particularly in the service industry (Murphy et al., 2018). When adopted in bundles, we believe that HR practices make employees realize that their goals align with their job and their organization. This happens mainly because adopting HR practices makes employees feel that their organization values them. The current study examined job security, promotion, autonomy at work, training/development, and communication as HR practices that collectively form a bundle. All these practices are directly beneficial for the employees. Due to their benefits, employees realize that their organization is on the same page as they are, which ultimately leads to a decrease in negative behaviors like TOI and job stress. This is also in accordance with Kooij and Boon's (2018) belief that the perception of HPWS increases congruence between employees and their organization, which results in positive outcomes.

The research sample was education sector employees. The research contributed to HRPs' literature by providing empirical evidence of the HPHRPs' effects in a new manner. The exploration added to the HRP literature by providing empirical proof of HPHRPs' recent impacts. The findings support all predictors of employee outcomes in the current study. The findings of assessing the mediating function of PO/PJ fits show that PO/PJ fits mediate HPHRPs and job outcomes (i.e., work stress/TOI) somewhat (but considerably).

Our investigation revealed a significant positive relationship between the PO fit and HPHRPs. This proves that HPHRPs share company values, goals, and aspirations with employees, energizing greater harmony between employees and groups (Boon et al., 2007). The current research has many useful findings similar to the ASA structure and past research (Boon et al., 2007). A sizeable portion, or 47.3%, of the variance in PO fit, was accounted for by HPHRPs. Takeuchi and Takeuchi (2013) stated that HPHRPs made up 28% of the difference in PO fit in Japan but reported that HPHRPs made up 29% of the change in PO fit in the Netherlands (Boon et al., 2007).

The outcomes presented in the present investigation indicate that HPHRPs shape the qualities and goals of employees, as HPHRPs caused major variations in the PO fit for employees in the Netherlands and Japan. The PO fit partially mediated the relation between HPHRPs and job stress/TOIs. Moreover, the PO fit had a similar effect on the two outcome variables. For job stress and TOIs, the change resulting from HPHRPs and the PO fit was 6 and 20%, respectively. The majority of the shift was brought about indirectly by HPHRPs *via* PO fit, indicating that PO fit is a crucial mediator in these links.

As far as the links between PJ fit and employee outcomes are concerned, the results are in accordance with our hypotheses and previous studies (Jin et al., 2018). With respect to PJ fit, the outcomes recommend that negative thoughts about the HR framework do not specifically make people consider leaving the association. It is also possible that such thinking influences employees' feelings of fitness with work, which is identified with their goal to quit. Similarly, it holds for the relations with job stress, which happen partially by means of PJ fit. This proposes that HRPs make it easier for employees to meet job necessities and be satisfied with their work in relation to their needs, thus diminishing their stress at work.

This investigation concentrated on PO fit, which is a popular and important fit type (Kristof-Brown et al., 2005). Compared to other forms of fit, PO fit has been shown to have a more solid association with employee outcomes (Kristof-Brown and Jansen, 2007). It has been discovered that a mismatch between a worker's abilities and the demands of their job leads to stress at work and intentions to quit. However, in this specific situation, other types of fit, such as PJ fit, may be significantly related to occupational stress and TOI (Chen et al., 2022; Pattanawit and Charoensukmongkol, 2022).

The findings of this study have significant practice-related ramifications. Generally speaking, achieving alignment between employees' and business values is vital if companies are motivated to improve employees' work experiences. The research demonstrated that HPHRPs, like training opportunities, elevated job security, promotion from the inside, and working independently, are compelling in this respect. Supervisors, in this way, should utilize the authoritative mission and objectives as the premise on which HPHRPs are planned. This will expand the arrangement of employee objectives and those of the association. Supervisors ought to exercise cautious thoughtfulness regarding the implementation and correspondence of HPHRPs to positively impact how employees see these practices. This will help strengthen employees' relationships with the association's way of life and reinforce their bonds with the association, which will thus make it more unlikely that they feel stressed and need to leave. Overall, this study contributed to the literature, particularly in the education sector, by identifying the ways in which TOIs and job stress can be reduced in the education sector. An important takeaway from this study is the crucial role of congruence. Employees show positive outcomes only when they think that their job and organization are aligned with them and are not moving in the opposite direction. One of the biggest lessons practitioners can take from this study is that they must adopt HPHRPs in the education sector, as it will make employees, whether they are nurses, doctors, or administrative staff, feel that their organization and jobs are in congruence with them. We can apply this concept of congruence to the whole society, especially in relationships. This means that individuals are more likely to show positive behavior when they believe they are fully aligned with others regarding goals, objectives, and much more. We tend to develop strong relationships with those who show higher congruency with us.

The discoveries of this examination ought to be interpreted in light of various constraints. For instance, the present study employed a cross-sectional design, and thus, no conclusions regarding causality can be established. For instance, it is conceivable that the degree of PO fit affects how employees see HPHRPs. Employees who successfully fit in with their organizations may have a favorable opinion of HPHRPs. Additionally, it is conceivable that individuals with low levels of work-related stress believe that their companies' opinions of them are predictable. More research with longitudinal or experimental designs is necessary to address the problem of causation. Second, common method bias may have inflated the associations since self-reported data from a single source was used. Third, it is unclear which set of procedures should be used to examine the correlation between HPHRPs and employee performance. The five practices used in the current analysis may not be an accurate representation of all HPHRPs used by companies; however, they need to be. In any event, the practices included in this analysis are among those that are most frequently used in studies linking HPHRPs and employee outcomes. Lastly, for the investigation, data were gathered from employees, and a convenience sample was utilized. Thus, the findings of the current study cannot be generalized to the current environment as a whole and are limited to the sample under consideration. Future studies might want to investigate whether the findings can be generalized across organizations and other geographical regions. Despite these limitations, our analysis confirms the significance of PJ fit and PO fits public links since the effects of HPHRPs depend on how well-employees get along with their companies. Fairness and social support have also been suggested as variables that may predict stress and turnover objectives (Leiter and Maslach, 2003). As per Leiter and Maslach (2003), employees feel distanced from the absence of value and support from their supervisors and colleagues, which, thus, may prompt negative results. Future researchers may wish to think about these relationships.

## Conclusion

In conclusion, this study provided valuable insights into analyzing how HPHRPs provide beneficial results. The impact of HPHRPs as significant drivers of employee outcomes *via* fits among employees in the education industry was well-supported by the findings of this study. The suggested links were tested in the education sector, an understudied area where research interest is growing. Thus, the work of this study strengthened the generalizability of ideas and policies in the countries examined. This is crucial since the investigation's contributing variables will significantly impact employees everywhere (Akhtar et al., 2022; Li et al., 2022).

## Data availability statement

The raw data supporting the conclusions of this article will be made available by the authors, without undue reservation.

## Ethics statement

The studies involving human participants were reviewed and approved by Ethics Committee of South China Normal University, China. The patients/participants provided their written informed consent to participate in this study.

## Author contributions

CY: investigation, writing—review and editing, and conceptualization. FY: investigation, methodology, software, formal analysis, and writing—original draft. Both authors contributed to the article and approved the submitted version.
